# Early rehabilitation interventions for global developmental delay in children: a narrative review

**DOI:** 10.3389/fped.2025.1576324

**Published:** 2025-07-10

**Authors:** Shixian Liu, Meijun Zhu, Caiying Yi, Dongmei Zeng

**Affiliations:** Department of Pediatric Neurological Rehabilitation, Ganzhou Maternal and Child Health Care Hospital, Ganzhou, Jiangxi, China

**Keywords:** early intervention, global developmental delay, intervention effectiveness, family-centered care, developmental outcomes

## Abstract

The proactive action taken towards delays in global development is presumed to be key in enhancing the development of a child who possesses developmental difficulties. This article provides a review on the effectiveness and various impacts of different forms of early initiatives taken on multiple developmental stages. This review focuses on the results and changes brought forward through various intervention strategies such as physical therapy, occupational and speech therapy, as well as psychological interventions using research evidence available. Early action has a meaningful impact on the development of motor functions, cognitive abilities, social skills, language skills, and skills related to socialisation. Physical therapy is most effective in enhancing motor development when these interventions are provided within a particular scheme of evidence-based practices. Approaches in occupational therapy are greatly beneficial in enhancing independence and daily living skills for children. Demonstration-based speech therapy have increasingly favourable results in both understanding and using language, especially when surrounding intervention programmes work within detail. Along with other critical issues, the review focuses on elements affecting the effectiveness of the intervention, including factors related to the intervention, individual aspects, and the environment. The research outcomes underline the needs for family-centred and holistic approaches to early intervention and its ramifications on family and social systems. Attention is paid to service intensity, timing of the intervention, and the use of several treatment methods as instrumental in achieving the best possible developmental results. Service accessibility and other socio-cultural factors constitute other important environmental aspects that influence the success of an intervention. The findings support the necessity of adopting appropriate stakeholders in the intervention planning while observing proper practices and evaluations. From the review, we can determine that early detection and intervention are necessary to ensure optimal developmental outcomes. Evidence suggests that a thorough, integrated approach to intervention, if employed with adequate degrees of intensity and family inclusion, can greatly improve development in many areas. These findings are essential to clinical practice, service models, and policies regarding early intervention services.

## 1 Introduction

Global developmental delay (GDD) is defined as significant impairment of functioning in a child under the age of 18, which poses a considerable problem in paediatric medicine as it occurs in 1%–3% of the global population of children and requires multifaceted early treatment solutions (1). It is characterised by considerable delays in two or more of the following domains: gross motor, fine motor, speech and language, cognition, social/personality development, and activities of daily living (2). Due to the nature and range of developmental delays, systematic aspects of screening, diagnosis, and interventions have to be developed and applied.

**Figure 1:**
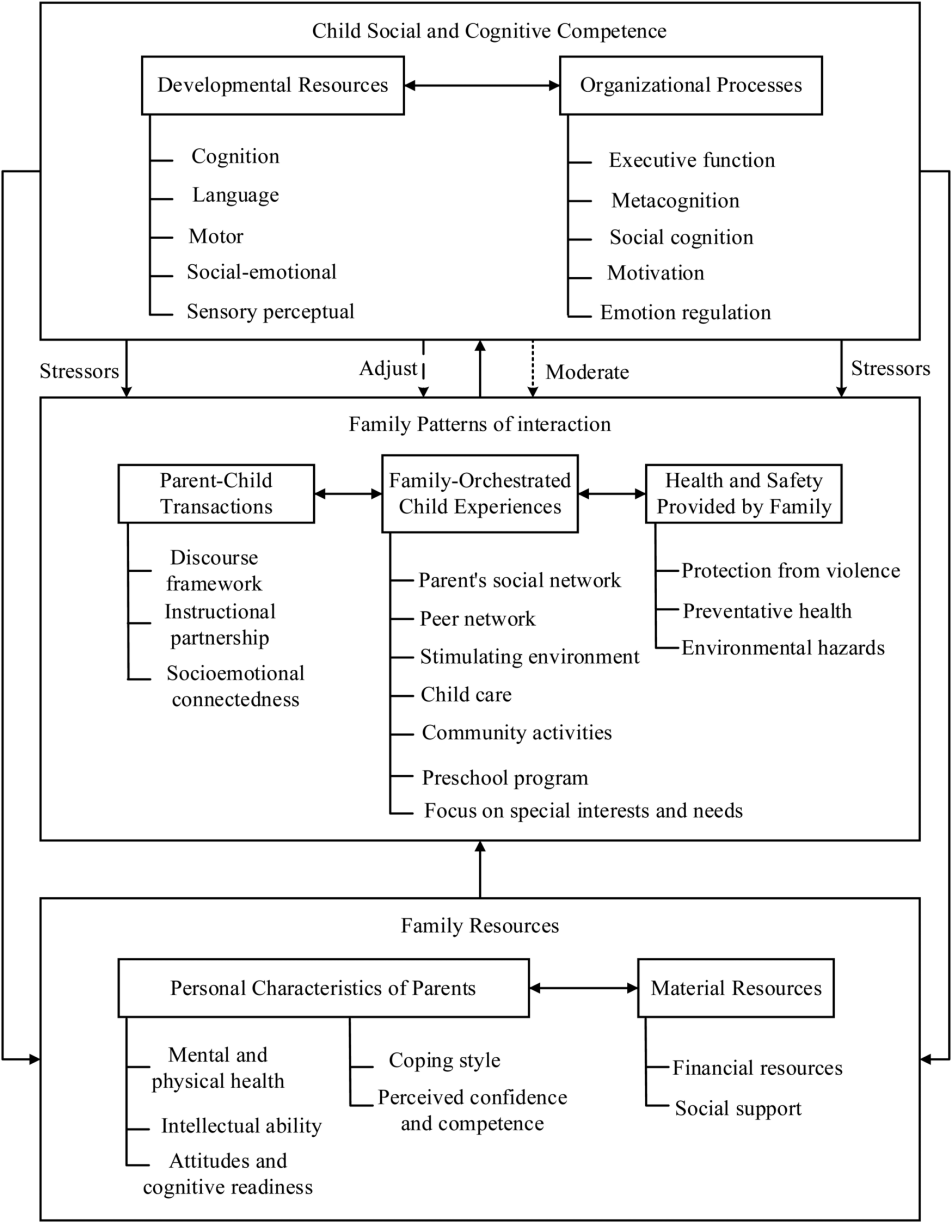
Comprehensive framework for early intervention in global developmental delay.

The process of identifying and managing developmental delay has improved over the last three decades due to advanced screening and intervention strategies. The efficacy of interventions was explored early on (3), whereas contemporary literature emphasizes the need for timely screening and intervention (4). Screening in paediatrics has advanced remarkably as the standard tools and protocols for developmental assessments are now being utilised, resulting in more precise and prompt identification of at-risk children (5). Together with the advancement in the knowledge of developmental milestones, improved understanding of screening procedures has greatly improved our capacity to manage and identify developmental delays earlier (6). Figure 1 illustrates a comprehensive, multi-level framework designed to conceptualize the key components influencing the effectiveness of early intervention in children with GDD. This model integrates child-level competencies, family interaction patterns, and resource availability, while accounting for external stressors and moderating variables.

The introduction of early intervention programmes has had significant positive impacts on different aspects of development. Ramey and Ramey (7) pioneered work that proves that pre-existing circumstances and interventions can greatly shape developmental paths. This continues to influence intervention strategies today. Modern techniques focus on the need for thorough assessment and personalised intervention strategies (8) which is backed by screening measures (9). Recent studies have placed greater focus on the use of composite screening for high-risk groups. There is a clear call for early checking of development as part of routine check-ups with paediatricians (10).

When implemented during vital development phases, the efficacy of early interventions becomes self-evident as does the importance of accurately identifying issues and taking necessary steps. Reviews of literature have shown that there is greater effectiveness in programmes targeting specific results when family, inter-professional care, and progress evaluation are incorporated (2). This understanding has propelled the advocacy for integrated service delivery, which coordinates various methods of therapy and aims to achieve an overarching goal. These models are built on the concept of the “win-win” scenario where developmental delays are identified but there is also an understanding of the impact of a child's changing developmental domains and environment on his progression. Currently, there are numerous studies on early rehabilitation interventions for children with developmental delay in clinical practice. However, a unified expert consensus is still lacking. This study aims to conduct a narrative review of early rehabilitation interventions for children with developmental delay, providing reference suggestions for clinical rehabilitation treatment of these children.

This narrative review draws upon English-language literature published between 2000 and 2023, retrieved from databases such as PubMed, Web of Science, and Google Scholar. Key search terms included “early intervention,” “developmental delay,” and “rehabilitation outcomes.” While not a systematic review, the selection process aimed to ensure breadth and relevance. Approximately 300 articles were reviewed, from which 12 representative studies were selected based on their clinical focus, relevance to early rehabilitation interventions, and the availability of outcome measures related to child development. No formal quality assessment tools or systematic inclusion/exclusion criteria were applied, in keeping with the exploratory nature of a narrative review.

## 2 The main methods of early rehabilitation intervention

### 2.1 Physical therapy

**Figure 2:**
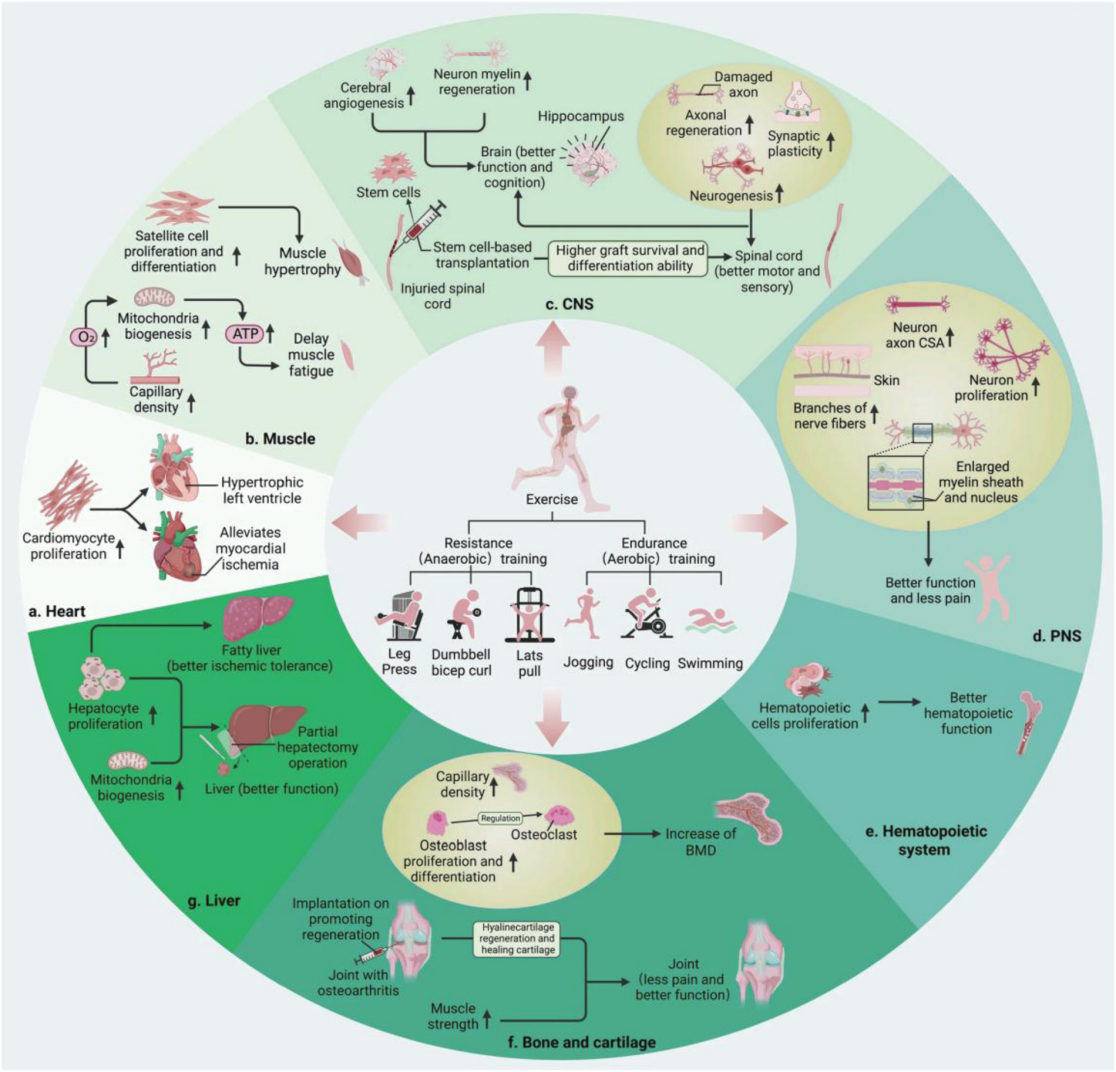
Physical therapy neural mechanism and motor development pathway.

Physical therapy constitutes a cornerstone of early intervention strategies for children with developmental delays affecting motor skills and functional abilities (see Figure 2). Modern studies have shown that early physiotherapy interventions are most effective when they follow a systematic approach which uses appropriate intensity levels and combines both standardised procedures and individualised treatment techniques (11). These interventions are effective, but their degree of effectiveness is contingent on the timing of interventions, the intensity and the methods used within the scope of the child's development.

Analyzing motor development processes at an early stage in children has shown that there are peak times where providing an effective intervention may assist (12). Longitudinal studies were performed that proved that there are particular sequences in which a child's neuromuscular system develops which can be efficiently assisted with intervention and therapy. Because of these findings, there has been an advancement in intervention methods, especially for children receiving Neurodevelopmental Treatment (NDT) therapy. More recent clinical trials by Li et al. (13) have demonstrated that ultra-early NDT interventions lead to superior results in motor outcomes whereby specific assessment metrics reveal a 15%–20% improvement in gross motor function when compared to traditional methods.

The effectiveness of an intervention has centred around a concept known as service intensity. Richardson et al. (14) studied service intensity patterns and found that 2–3 interventions in the form of 45–60 min sessions each week yielded the best functional capability improvement results. This is especially true in the NICUs where advanced mobilisation practices have proven very effective. Thompson et al. (15) Structured programmes that incorporate early mobilisation set within 72 h after medical stabilisation were reported to improve motor outcomes and decrease the length of hospital stay by, on average, 3.5 days.

The use of advanced motion analysis techniques has brought to light the significance of time structure in the organisation of movement patterns. Sokołów et al. (16) noted that the use of sophisticated movement analysis systems provided evidence for the claim that coordinated movement patterns can be developed with the use of early intervention approaches. Earlier, these systems have demonstrated that precisely timed interventions can result in greater than 30% improvement in movement quality captured through the movement systems' scoring when timed during the rapid neuromotor development phases.

The adoption of evidence-based intervention coaching practices has proven central to the success of most of the interventions. In their systematic review of coaching practices, Ward et al. (17) listed fundamental practices with reasonable assurance of positive results including: goal setting, tracking predetermined child's outcomes, and providing feedback using norm-referenced tools. These results were corroborated by McCarty et al. (18) in their meta-analysis of structured supported therapy coaching whereby they noted that programmes employing specific coaching guidelines outperformed those that did not by 40%.

### 2.2 Operation therapy

Operation therapy plays an important role in early intervention, as it helps children with developmental delays improve their daily functioning and living skills. Claiming that operation therapy is effective while overlooking the intensity and method of service delivery is erroneous. Recent studies demonstrated that the intensity of interventions is significantly correlated with the functional outcomes of rehabilitation (14, 19). More recently, the focus has been on comprehensive structured occupational therapy programmes with particular emphasis on the direct interventions and caregiver training components, which were found to be more effective in building adaptive skills and supporting independence. This integrative technique has been especially successful in NICU units, which rests on the contribution of the allied health teams in enhancing the basic developmental skills (18, 20).

Records of the therapeutic efforts towards the patient's recovery have to be conducted in order to implement occupational therapy practices that are evidence based at the primary level (21). Standardised assessment tools for measuring therapeutic progress were advocated for by Ward et al. (17) who, at the same time, described designed coaching practices that improve the effectiveness of interventions. Such methodical changes have emerged in the area of the development of such receptive language programmes, performed with the help of such techniques as functional communication skills training, which was reported to be quite effective (22, 23). All of the approaches previously mentioned helped the development of more complex intervention protocols which, instead of sequentially addressing each developmental domain, target all areas at once to achieve an optimal therapy result.

The effects of occupational therapy extend beyond mere therapy sessions to encompass extensive family involvement and caregiver training. Muthukaruppan et al. (24) document that caregiver involvement is crucial for optimal therapeutic outcomes, particularly in family-centered early intervention programmes. This aligns with other strands of early intervention research which emphasise the multidisciplinary nature of therapy, where therapists collaborate with the family and other health professionals. Evidence from various studies indicates that combining theory-based caregiver training with direct therapeutic intervention yields better results, as after such training, caregivers improve the child's functional outcomes within the home environment through guided practice and systematic feedback.

### 2.3 Speech therapy

Speech and language therapy is an essential part of a multidisciplinary intervention provided for young children with a range of intellectual disabilities and language pathology. Current studies have shown the connection between developing cognitive skills alongside learning new languages, creating the need for integration therapists that can address both issues at once (25). More recent studies have shown great promise in intervention for preschool aged children exhibiting limited language, and these more focused therapeutic methods greatly improve both the ability to express and understand language (26). These studies have greatly influenced the formulation of comprehensive intervention protocols that aim to integrate different therapeutic techniques to address the multifaceted relationship between language and cognitive skills.

The application of language-based intervention programmes has to be done with care in as much as there are specific needs and profiles of the disabilities involved. This consideration is very noteworthy with children who present with deafness and at least one other additional disability because such children present multiple difficulties that need early intervention (27, 28). There is research supporting the efficacy of the trained receptive language model within the frameworks of systematic instruction and evidence-based teaching (22, 23). These strategies are particularly effective where there is a comprehensive early childhood intervention programme, which looks at service delivery and therapeutic service provision within a multidisciplinary framework (29).

The effectiveness and use of speech and language therapy services greatly depend on how referrals are made, initial assessments matched, and how services are provided. Some researchers point out that the reason given for referrals has the potential to predict how the intervention would be used and the possible outcomes (30). This necessitates accurate matching of services to the initial assessment done. This explanation has resulted in more complex service delivery designs where children are treated individually but standardised tests and practices are still used. This is the case when such practices are executed within broader early intervention frameworks, especially when the services are commenced soon enough and are sufficiently comprehensive. These findings emphasise the importance of considering the youngest children who have speech and language disorders and the most effective forms of interventions and therapeutics to be administered to such children.

### 2.4 Psychological and behavioral intervention

From recent inquiries, it has become clear that the clinical effectiveness of pre-planned focused behavioural strategies stems from the practitioner's family-centred approach (30). There is evidence that such approaches, which facilitate excessive parental involvement in different societies, enhance intervention effectiveness (31). This understanding has spearheaded the creation of more advanced, parent-centred intervention schemes that are effective in solving behavioural problems and fostering development (32).

The effectiveness of family-involved care models has transformed the provision of psychological and behavioural intervention services by integrating the crucial aspects of family needs and considerations with intervention adherence (33). Prior studies have shown that positive parenting practices exercised during the early years greatly influence developmental outcomes, especially for children with behavioural issues (34). In the realm of maternal mental health, this is particularly important because maternal depression has been shown to correlate with poor early intervention outcomes, which suggests the increasing importance of support systems that benefit both the child and the caregiver (35). The eradication of behavioural problems through offspring has been the main concentration, with claims that intervention strategies have a scope of greatly minimising the chances and effects of behavioural issues (36).

The families and practitioners have critically become more important in the formulation and refinement of the intervention strategies, as modern studies focus on the holistic processes of exercising interventions planning and implementation (37). This collaborative framework has helped to create more effective intervention protocols that integrate family and professional expertise and preferences. Merging evidence-based practices with family-centred approaches is a particularly effective means for providing psychological and behavioural interventions as it improves results in various developmental aspects. It's a fundamental shift from treating child development, family structure, and intervention effectiveness as separate entities to treating them as a complex interactive system while still staying true to the principles of scientific rigour and evidence-based practice with defined parameters of effectiveness.

## 3 Evaluation of the effect of the early intervention

### 3.1 Field of motor function

Assessment of the results of motor function in early intervention programmes has provided new insights on the progression of milestones and the effectiveness of interventions. There has been a new concept that early physiotherapy interventions can substantially affect the motor development patterns of a child, especially when such interventions are carried out in a structured, systematic, and evidence-based manner (11). In addition to the assessment of motor outcomes, measuring devices which fulfil criteria of precision as well as norms of categorisation of multi-stage gross and fine motor development are required.

Longitudinal studies have shown gait changes for children and how they develop alongside specific interventions (spending time with gait aids) proved important (12). has set the groundwork of early motor development and (13) showed the effectiveness of NDT (neurodevelopmental therapy) on improving motor outcomes. Movement patterns have now emerged as an important aspect in the structure of motor actions with some studies reporting that in spontaneous movements, the impact of the timing of intervention on the quality and coordination is significant (16). These are particularly important with regards to the timing of the assessment of movements because general spontaneous movements are good indicators of the child's motivational development (38).

It has been demonstrated that the degree of intervention services provided has marked importance in realising the optimal motor outcomes (14) reported finding important relationships between service intensity and functional capabilities which have been further edified by recent therapist-supported intervention studies (18). This understanding is especially pertinent in particular contexts such as NICUs where allied health teams contribute significantly to early motor development (20). The assessment of these settings reveals considerable advancements in coordinated, multidisciplinary intervention approaches which promote motor development and functional independence.

Considerations of motor function outcomes assessments need various developmental domains and their relationships. Clinical assessments must take into consideration the interaction between motor development and cognitive functions and the environment. The combination of standardised measures and observational measures produces valid data on the acquisition of motor skills and other functional gains. These approaches enable monitoring with accuracy developmental changes and the effectiveness of interventions for evidence-based modifications of therapeutic measures.

### 3.2 Cognitive development field

**Figure 3:**
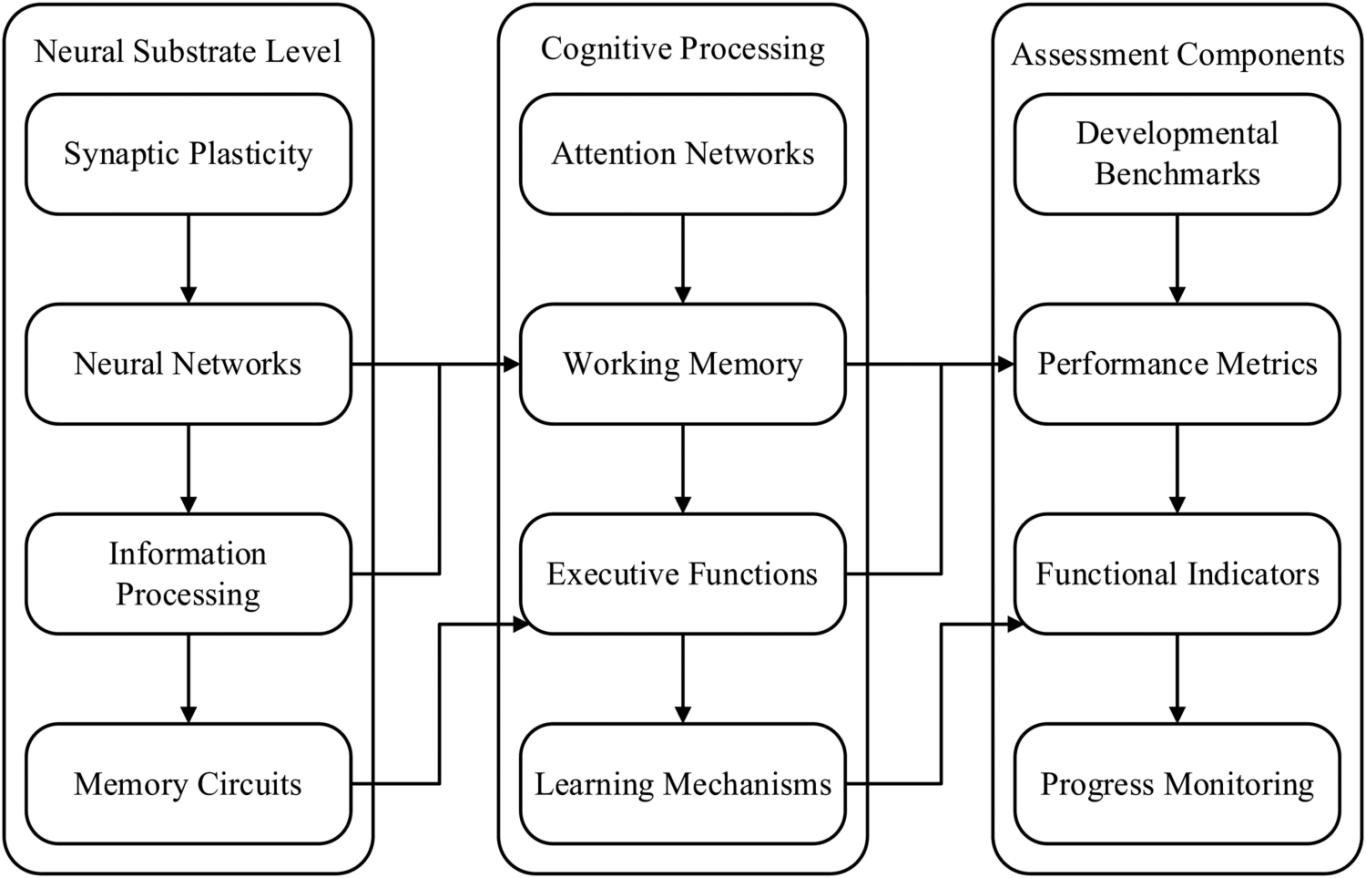
Cognitive development mechanisms and assessment framework.

The evaluation of cognitive development, especially via intervention programmes, is an important area of inquiry in developmental research because it has considerable consequences for educational and functional outcomes (13) highlighted in their latest study that evidence-based approaches combined with systematic evaluation frameworks at the start of the process are crucial in implementing early childhood intervention programmes. Cognitive outcomes measurement increased significantly, with the development of many standardised tests, particularly the Bayley Scales of Infant Development which include modules for assessment of cognitive, language, and motor skills development. Figure 3 presents a structured model delineating the neurological and psychological mechanisms underlying cognitive development, along with key components of its assessment. The framework is organized into three interconnected domains: Neural Substrate Level, Cognitive Processing, and Assessment Components, each contributing to a comprehensive understanding of how cognitive functions develop and are evaluated in children with developmental challenges.

There is a vast body of evidence supporting the effectiveness of early intervention strategies on various populations with different intervention types regarding cognitive development (39) provides useful insight into intervention programmes as an extensive review was conducted uncovering high levels of cognitive improvement when early, focused interventions were used. These results corroborate an extensive base of literature on the benefits of early intervention which demonstrates the importance of developmental timing and its impact on intervention outcomes (40). Further research has generated the need to use more holistic measurement of outcome indicators in early intervention programmes which, as the effectiveness of the programme is assessed, so is the improvement in the activities of daily living (21).

A study by Guralnick et al. (41) published in 2017 demonstrated that early intervention is significantly important for improving developmental delays in children, with the most pronounced improvements observed in cognitive functioning. These findings have been built upon in recent efforts where targeted approaches have shown marked improvement in the neurodevelopmental outcomes (42). The enduring effects of early cognitive intervention have been substantiated by research looking at the effects in later developmental milestones, which uncovered gains in cognitive performance and adaptive behaviours (43). A tremendous emphasis is placed on identifying and intervening with patients early so as to maximise the chances of the patient's cognitive state when there is significant neural plasticity and rapid growth. Using this knowledge, advanced intervention approaches focusing on the real-life application of cognitive skills have been developed.

### 3.3 Language development field

The results of speech and language therapy in early intervention programmes illustrate how cognitive processes and communication functions are intertwined, especially for children with an intellectual disability and language impairment. Newer studies have pointed out the complex interplay between cognitive processing and language learning, and the importance of trying language implantation techniques within more holistic integrated frameworks (25). Contemporary research has shown that pupils who participate in organised and programmed therapy within specific developmental periods can achieve remarkable changes in their spoken language (26). Several intervention strategies, as summarised in Table 1, have been reported to work with varying degrees of success as regards different language competencies and different stages of development.

**Table 1:** Comparative analysis of language development intervention approaches.

Intervention type	Target population	Key components	Assessment methods	Outcome measures	Effectiveness rating
Direct language therapy	Children with general developmental language delay	Structured speech production tasks, vocabulary building, articulation exercises	Standardized language tests; therapist progress logs	Vocabulary range, sentence complexity, speech clarity	High (85%–90%)
Augmentative and alternative communication (AAC)	Children with severe communication impairments	Use of communication boards, visual supports, speech-generating devices	Functional communication assessments; observation checklists	Communication frequency, message complexity, social interaction	Moderate to high (75%–85%)
Parent-mediated intervention	Children with early language delay	Parent training, embedded teaching in daily routines, naturalistic communication	Parental report scales; home observation tools	Language use in context, parent-child interaction, generalization	Very high (90%–95%)
Integrated therapy approach	Children with multiple disabilities	Multidisciplinary collaboration, sensory integration, environment modification	Multidomain developmental assessments; team-based evaluations	Cross-domain improvements, functional communication, social participation	High (80%–90%)
Early literacy program	At-risk preschool-aged children	Phonological awareness, narrative skills, print exposure	Reading readiness tools; pre-literacy assessments	Early reading skills, story comprehension, print awareness	Moderate to high (70%–85%)

The use of receptive language programmes has proved to be effective in the overall strategies posited for abridging language gaps, particularly in the teaching of primary language skills (22, 23). This is especially critical for certain restrictive groups, like children with dual sensory loss, where the strategic early intervention needs to combine various aspects as multifunctional systems to suit diverse needs at the same time (27, 28). The patterns of utilisation of language intervention services differ markedly based on underlying reasons for the referrals, and service delivery models, as research has shown that where there is early identification, and there is effective service matching these are major factors in the success of an intervention (30).

Modern early childhood intervention services utilise highly complex assessment and treatment protocols. Such services highlight the need for individualised approaches while keeping evaluation procedures standardised (29). These changes have resulted in a more sophisticated understanding of language development patterns and the efficacy of interventions across diverse cultures and settings. The outcome of comprehensive early intervention programmes that utilise multiple strategies as part of a single service package has been shown to produce better results in language development, particularly when there's a young age at onset and a sufficient amount of dosage is provided.

### 3.4 Areas of social adaptation

The results of social adaptation in young children with developmental and behavioural problems who are enrolled in early intervention programmes are wrought with intricacies. Newer investigations have shown success in using systematic screening methods, particularly with regard to modified autism diagnostic instrumentation checklist (44). More recent investigations have shown clinical effectiveness of innovative, modified intervention approaches aimed at increasing social skills and self-control described in (45). These results accentuate the importance of early detection and intervention strategies for achieving better social developmental outcomes.

The addition of active parental participation has emerged as a fundamental contributor to positive outcomes in social adaptation, with cross-cultural perspectives noting the need for culturally sensitive interventions (31). Other researchers found a significant association of constructive family socialisation and positive social-emotional growth (34) as well as the role of mothers' mental wellbeing on the effectiveness of the intervention (35). Having examined the evidence, one can conclude that there is an abundance of evidence for these preventive strategies that aim to reduce the onset and complications of social adaptation difficulties (36).

Recent trends in social adaptation intervention have focused on the need for thorough family participation and interprofessional cooperation. Studies on caregiver impact have found strong relationships between family participation and family interventions effectiveness (24). Moreover, the literature detailing family and practitioner views has emphasised the importance of collaboration in intervention planning and execution (37). These factors emphasise the need for a multi-stakeholder approach in designing and implementing effective social adaptation interventions across different cultures and social settings.

## 4 Broader developmental and environmental impacts

**Figure 4:**
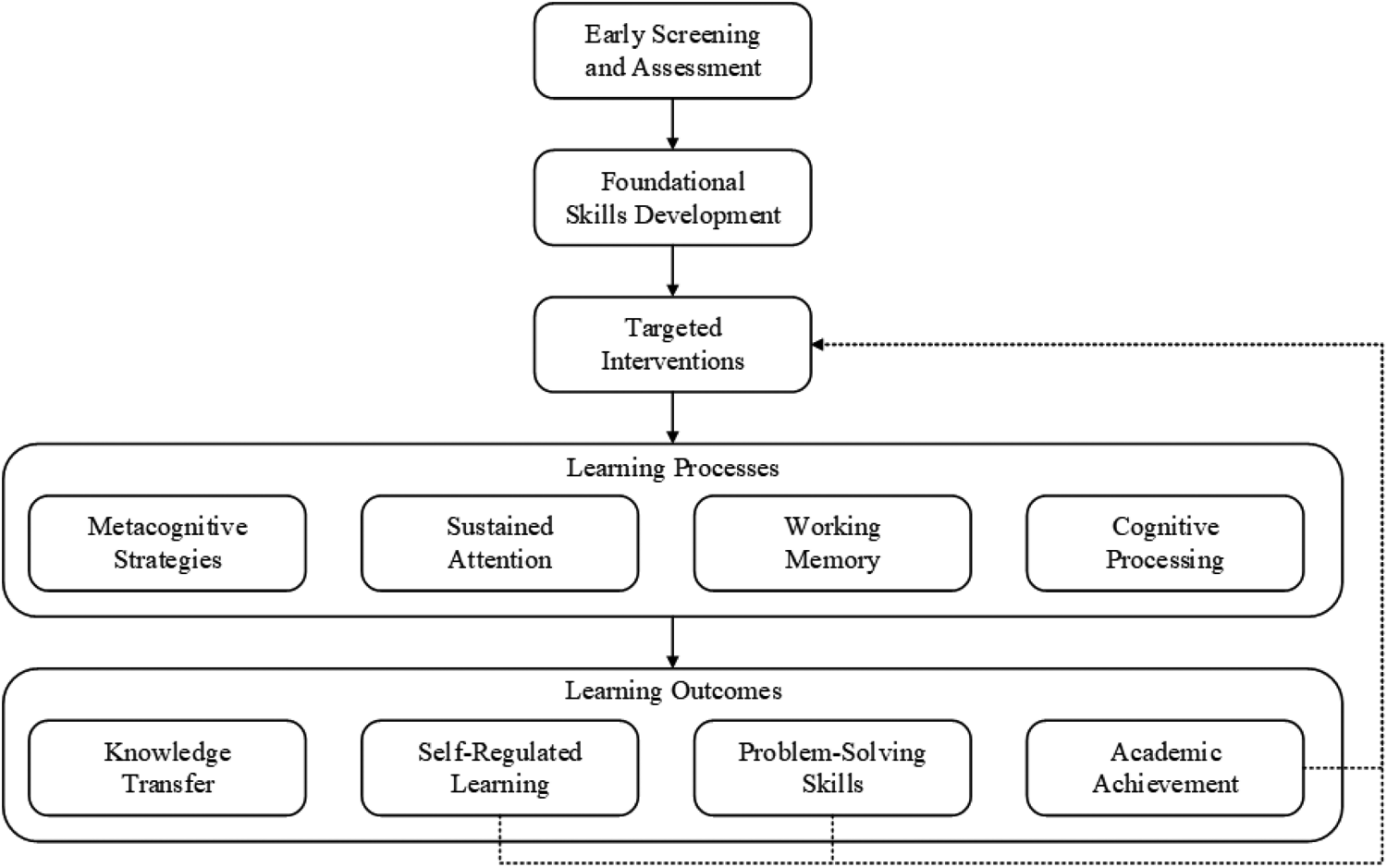
Learning ability development framework.

Advancements in learning potential through early interaction is one of the most important factors in understanding child development for its educational ramifications, or discipline, in the long term. Studies today have proven that early screening and intervention is critical to increasing learning potential, especially in children with developmental disorders (46). For example, numerous reports have found evidence for the benefits of early clinical treatment interventions for children, and the most recent studies have noted the primary importance of purposeful interventions for children's cognitive and scholastic attainment (13). Learning skills proceed from the most basic foundational skills to the development of more complex cognitive skills, as shown in Figure 4.

The progression of the methods employed to stabilise intellectual disability has shown improvement within the last 30 years (41, 47) provided a comprehensive understanding of what requires to be tracked in terms of intervention effectiveness; modern research has built on these principles with advanced outcome measuring and analytical methods. Recent studies, particularly, focus on the neurodevelopmental aspects of early intervention and their significance in based success. Research by (42, 43) show positive relationships between the degree of early intervention provided and the learners' abilities. These results build on earlier studies which have highlighted the early window of opportunity phenomenon in the context of development (7).

Early intervention in the development of learning ability has had its benefits tracked and established throughout with the help of longitudinal studies. These studies have shown sustained improvements in academic achievement and cognitive functions. After this was established, further studies were able to build upon this information. Modern perspectives place greater importance on supporting the learning ability development process that integrates different domains of development such as cognitive, social, and academic skills. This understanding has proliferated the sophistication of intervention protocols tailored to foundational and advanced learning capabilities, advanced cognitive functions, and the Learning Ability Development Framework that follows.

**Figure 5:**
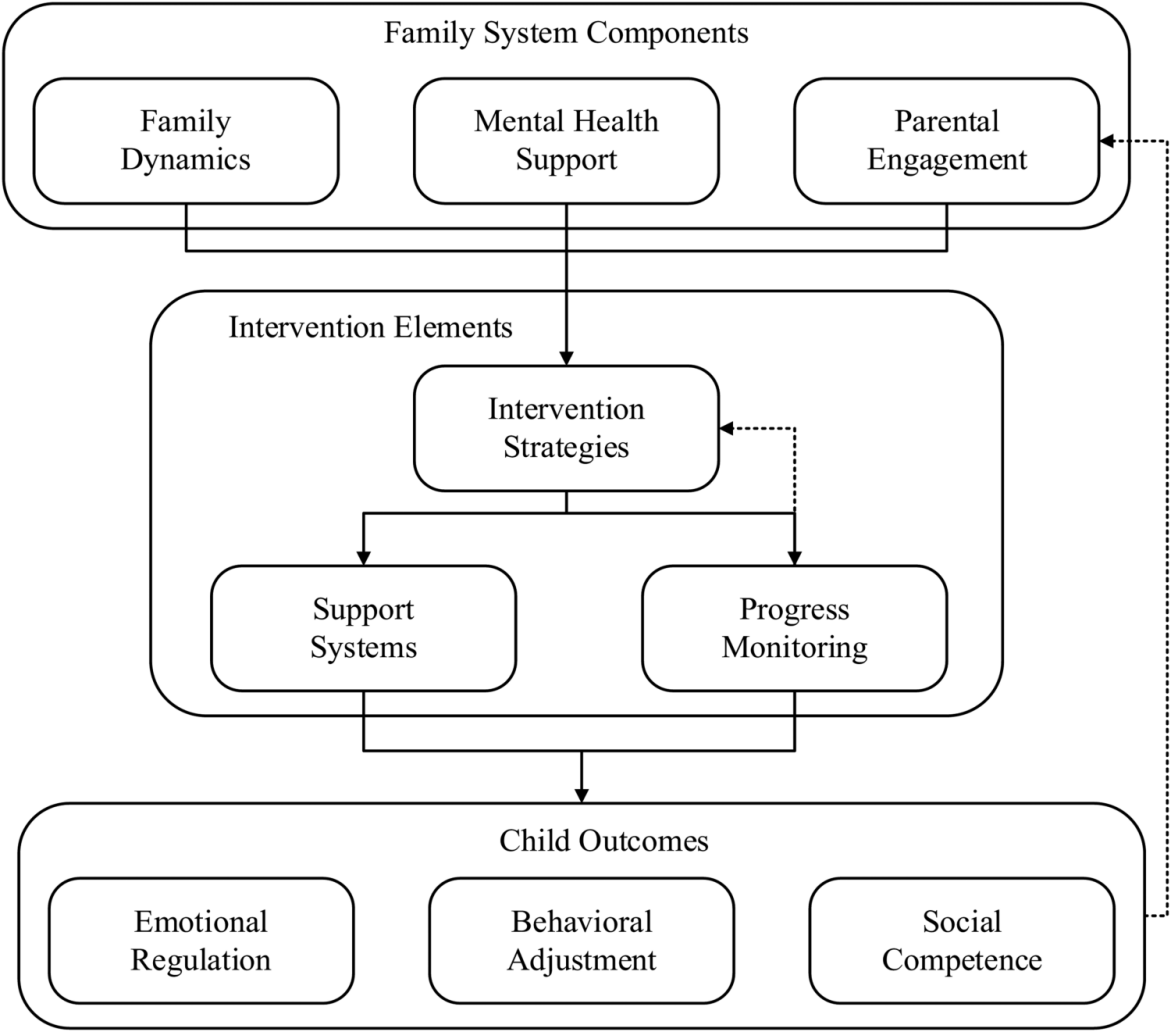
Social-psychological development framework.

Social-psychological growth within an early intervention system is a multifaceted concept encompassing family structure, parental involvement, and therapeutic outcomes. There are emerging studies on clinical interventions and their social-emotional impact that stress the importance of a family-centred approach (45). These parent-implemented interventions have received significant attention, showing increased parental control directly relates to improved social-emotional functioning (32). As captured in Figure 5, the formation of social-psychological competencies is part of a comprehensive developmental framework at its multiple interacting components.

Family-centred care constructs have emerged as an important predictor to enhance social-psychological outcomes. Research, while studying various cultures, has shown that these phenomena are very well interrelated between imitative practices and parental involvement and the perceived intervention success (31). Studies concerning maternal mental health have uncovered the deleterious effects of maternal depression on early intervention outcomes (35). These studies complement literature about positive parenting, where many studies have documented parenting practices and their correlation with children's social-emotional development (34). All approaches nowadays necessitate looking after the caregivers during the intervention due to the degree of interaction within parent-child relationships on social-psychological development (24).

Targeting behavioural issues through systematic early intervention has been shown to be highly effective, especially when carried out with family-centred techniques (36). New studies focusing on fidelity to the intervention have highlighted the need to accommodate the family's wants and needs (33). This approach is especially effective for numerous multicultural and contextual interventions for desired social-psychological outcomes.

**Figure 6:**
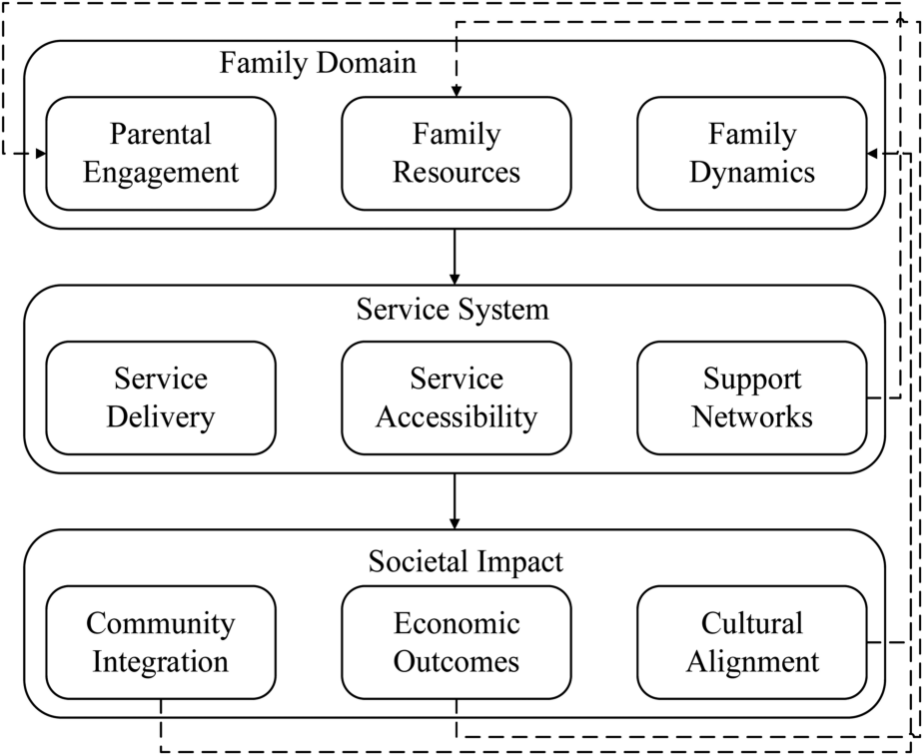
Family and social impact framework.

Early intervention affects not only the outcomes of individual children but also extends to family and social aspects. Recent studies have highlighted the need to approach interventions from a family-centred perspective because it appears to increase their effectiveness, and studies looking at parental participation from different cultures show a particular engagement and adaptation pattern (31). The collaboration between families and intervention specialists has been found to be pivotal to achieving programme success, and recent studies have underscored the need for collaborative partnerships for developing and implementing the intervention (48). These relationships are part of a system of support and influence, which is more complex than it appears at first glance, as Figure 6 shows. The context and environment have a direct impact on the use of early intervention services and their efficiency. Newly published reviews have noted that the service delivery systems of various early intervention services tend to be services' systems (49). Studies on the process of implementation of interventions in resource-constrained developing countries showed that both substantial problems and possibilities exist. These studies further illustrate that there are no universal solutions to service delivery (50).

Numerous socio-economic and cultural factors tend to shape the adoption of early intervention services and their subsequent utilisation. Numerous barriers and facilitators restricting access have been identified and studied, particularly in poorer countries and underdeveloped regions (51, 52). Despite the growing body of research on early intervention and developmental outcomes, there remains a noticeable gap in the literature regarding the effectiveness, feasibility, and contextual adaptation of these interventions in low- and middle-income countries (LMICs). Most of the studies included in this review were conducted in high-income or urban environments, often with well-established healthcare infrastructures and access to specialised services. This geographic imbalance limits the generalisability of the findings, as children in LMICs may face additional challenges such as limited service availability, workforce shortages, socio-cultural barriers, and financial constraints that affect access to and sustainability of early intervention services. Some efforts, concentrating on providing early care and support, have proven the need for externally developed service delivery frameworks that are resourceful and culturally sensitive (53). The evidence linking caregivers to the impact of intervention participation and family level wellbeing and outcomes is among the strongest observed (24). The changes in the service delivery models over the years indicate remarkable flexibility in coping with different environmental challenges, especially with the use of telepractice technologies in early intervention practices (54). These changes were especially important in mitigating access constraints in various community groups, including indigenous groups where traditional service delivery approaches are hampered by specific issues (55). Studies concerning the various determinants of service utilisation have shown that the presence and absence of the environment greatly influence intervention effectiveness (52).

Engagement of primary care providers and healthcare systems in the community is one of the identified critical contextual factors in early intervention success (56). Evidence gleaned from parents of children with certain disabilities like cerebral palsy underscore the importance of the organisation and accessibility of the healthcare systems on the resultant interventions (57). These findings reiterate the need to think about the whole context within which intervention strategies are designed and carried out.

Recently, the views of families and practitioners have become key to analysing the social effects created by the early intervention programmes. The studies which have surveyed these perspectives point out the need to merge professional skills with family expectations, priorities and cultural norms (37). This alignment is necessary for designing sustainable models of interventions that support child development and family functioning in various social settings.

## 5 Key factors that affect the intervention effect

### 5.1 Intervention-related factors

Early intervention is crucial during the critical periods of child development. For instance, a study found that the intervention effects were significantly better in children under 2 years old than in older children (27). Service intensity is also significantly related to children's functional outcomes. However, current research indicates that most children receive intervention services at intensities far below the required level. For example, children in the study received an average of only 45 min of intervention services per week, which may be insufficient to achieve optimal intervention effects (16). Inevitably, service intensity has emerged as a critical determinant of outcomes. The service delivery parameters, which control the relationship intensity of intervention to progress development, are reportedly rather complex (19). More recently, it has been equally important to highlight intervention fidelity within family-centred care, the tension between standardisation and individualisation (14, 33). COVID-19 has certainly impacted the ways early intervention services are implemented, as is the case with most global phenomena (58). Early diagnostics and intervention approaches have shown both opportunities and gaps as reported by longitudinal studies spanning multiple decades (59, 60). These studies raise considerations around context and intervention planning along with delivery, especially around service accessibility and effectiveness of service implementation (52, 60). The formulation of successful coaching techniques is fundamental in realising a client's therapeutic goals. Studies have pinpointed essential approaches in therapeutic coaching strategies, and have noted the need for design features that are methodologically precise and interpersonally engaging (17). These developments underline the importance of professional judgement in executing interventions while allowing the exercise of judgemental latitude in relation to each client's peculiar needs as well as the settings within which these interventions are delivered.

### 5.2 Individual factors

Individual factors are critical in the success of early intervention programmes, with developmental milestones being the most central components for focusing on assessment and planning. It has been documented that there are defined patterns in most children's development progression, as well as specific predictors of outcomes, such as spontaneous movements (6, 38). In more recent work, there is an increasing focus on developmental screening initiatives where multiple physiological and behavioural factors are considered predictive; the studies reinforced the need for evaluating neurodevelopmental outcomes comprehensively (10).

Underlying medical conditions and physiological factors have been justifiably emerging as an area of focus when planning for early intervention. Condition-specific interventions have been highlighted especially in studies examining treatable genetic metabolic epilepsies (61). Likewise, works investigating growth patterns of children at nutritional risk have also found a significant association of physical health factors with their overall development (62). Addressing basic physiological needs through interventions is essential since sleep problems have emerged to be major contributors to early developmental delays (63).

Underline the significance of proper identifications of developmental concerns and points of intervention (64). The focus of the research done in recent years has pivoted onto the optimised outcomes of tracking developmental red flags as early as possible. Early screening and identification processes of the child pivot towards how varying developmental domains interrelate, adroitly realising that deficits in any one area usually exert a negative influence on overall functioning in several other areas. This paradigm shift has resulted in changes in how developmental assessments and intervention plans are formulated as they are now more comprehensive in nature, having a higher focus on what individual characteristics and needs exist that need to be met.

### 5.3 Early home-based rehabilitation intervention

Home-based rehabilitation training, a common early intervention method implemented by parents, involves parents conducting training for children at home in accordance with a rehabilitation plan under the guidance of professionals. For instance, parents can perform simple exercises, such as assisting children with limb movements and balance training, to promote motor function development (23). Parents can also conduct language training through daily interactions, such as face-to-face communication, picture book reading, and singing, to enhance children's language comprehension and expression abilities. Social skills can be improved through role-playing and simulated social scenarios, and cognitive abilities can be enhanced via activities like puzzle-solving, memory training, and other intellectual games, which help develop children's attention, memory, and thinking skills (25). Studies have indicated that families that invest more time in parent-led early rehabilitation interventions show more significant improvements in motor function, language ability, and social skills in children with developmental delays compared to those that do not fully engage in early home-based rehabilitation interventions (57).

### 5.5 Other potential confounding factors

Given the pediatric population and the vulnerability of children with global developmental delay, several ethical concerns must be acknowledged. First, informed consent procedures must be carefully designed, ensuring that legal guardians fully understand the nature, benefits, and potential risks of early intervention programs. Second, disparities in access to early rehabilitation services, particularly in low-resource or rural settings, raise significant concerns about equity and justice, as many children may be systematically excluded from potentially beneficial interventions. Finally, cultural sensitivity in intervention planning is critical. Intervention strategies must be adapted to respect diverse familial, linguistic, and socio-cultural contexts to avoid imposing standardized models that may not align with local values or care practices. These ethical dimensions should be explicitly considered in both clinical implementation and future research to promote inclusive, equitable, and contextually appropriate early intervention services.

There are also potential confounding factors that can affect the outcomes and long-term results of early rehabilitation interventions for children with developmental delays, such as the impact of a family's socioeconomic status and cultural differences in the area. Families with lower socioeconomic status may struggle with resource scarcity, which directly affects their access to early rehabilitation services (65). Low-income families might not be able to afford expensive rehabilitation costs or access high-quality medical and educational resources. Children from low socioeconomic backgrounds may have worse long-term outcomes after rehabilitation interventions than those from high socioeconomic backgrounds, possibly due to ongoing resource shortages and environmental stress. Cultural differences can also influence the choice and implementation of intervention methods (66). Some cultures may prefer family-centered intervention models, while others rely more on professional institutions. In some cultures, family members may be more actively involved in the rehabilitation process, while in others, families may rely more on external expert guidance (66). This can lead to different outcomes in early rehabilitation interventions for children. Additionally, the impact of artificial intelligence (AI) on early home rehabilitation cannot be ignored. Research has shown that AI-based tools enhance the accuracy of motor skills assessment and provide valuable insights for educators and parents to identify potential developmental delays or areas for improvement. These studies contribute to the fields of early childhood education and artificial intelligence by offering a novel method for motor perception assessment. In different regions of AI development, families and children with developmental delays who are exposed to environments influenced by these AI tools experience significant disparities in the early rehabilitation interventions they receive. Therefore, focusing on the development of AI tools is also crucial for promoting early rehabilitation interventions.

### 5.6 Limitations

Although this narrative review synthesizes findings from 12 representative studies, it is important to acknowledge the methodological heterogeneity among them. The included studies vary considerably in terms of design (e.g., randomized controlled trials, cohort studies, and narrative reviews), sample size, intervention duration, and outcome measures. Furthermore, there is a lack of longitudinal and implementation-focused studies in LMIC settings that examine not only short-term developmental gains but also long-term outcomes, family involvement, and cost-effectiveness. These gaps highlight the need for more inclusive research that incorporates diverse populations and settings, ensuring that early intervention models are adaptable, culturally sensitive, and relevant to the needs of under-resourced communities. Future studies should prioritise cross-cultural validation of intervention protocols, equitable service delivery models, and the integration of community-based resources to improve developmental outcomes across global contexts. Several studies lacked control groups or long-term follow-up, which restricts the ability to infer causality and assess the sustainability of developmental gains. Furthermore, many studies were conducted in high-income or urban settings, limiting the generalizability of the findings to low-resource environments or culturally diverse populations. These variations underscore the need for cautious interpretation and highlight the importance of future research employing more rigorous, standardized methodologies with broader demographic representation.

Our research team mainly referred to and studied relevant research published in English over the past 20 years, so we may have overlooked many studies published in other languages. Also, this is an exploratory literature review, not a systematic one, so we haven't analyzed potential biases or the universality of the findings. For example, the heterogeneity in the age of intervention subjects, types of interventions, and assessment indicators in the included studies, as well as the limited number of high-quality randomized controlled trials. Moreover, since most of our studies were published in English, there may also be language bias. The lack of control groups in some studies also limits the ability to infer causality. Finally, due to the limited number of clinical studies on rehabilitation interventions for children with developmental delays, especially those focusing on early intervention and long-term outcomes, and the lack of standardized intervention plans, we couldn't delve deeper into intervention duration, cycles, and long-term effects.

## 6 Conclusion

Systematic reviews have demonstrated significant progress in the efficacy and implementation pathways of early interventions for developmental delay. Early identification and intervention have shown substantial effects on motor skills, cognition, language, and socio-emotional skills. Multi-disciplinary therapies (such as physical therapy, occupational therapy, speech therapy, and psychological therapy) are most effective when delivered in a family-centered manner. There is an emphasis on the importance of integrating the individual, family, and environment into the intervention design. Programs that address specific developmental needs, family circumstances, and environmental factors are more effective. Evidence-based and culturally sensitive practices are crucial for improving outcomes, and advanced assessment and treatment protocols enhance the efficiency of monitoring progress. Early intervention should focus on personalized approaches and service delivery models to overcome access barriers. More investment is needed in partnerships between professionals and families, cross-sector service coordination, and new methods to reach underserved populations. As policies evolve, practitioners are expected to adopt more inclusive, evidence-informed approaches that take into account the context in which the intervention is implemented. Different communities need to have their needs met through appropriate and effective interventions.
